# Ethanol Distribution, Dispensing, and Use: Analysis of a Portion of the Biomass-to-Biofuels Supply Chain Using System Dynamics

**DOI:** 10.1371/journal.pone.0035082

**Published:** 2012-05-14

**Authors:** Laura J. Vimmerstedt, Brian Bush, Steve Peterson

**Affiliations:** 1 National Renewable Energy Laboratory, Strategic Energy Analysis Center, Golden, Colorado, United States of America; 2 Peterson Group, West Lebanon, New Hampshire, United States of America; Philipps-University Marburg, Germany

## Abstract

The Energy Independence and Security Act of 2007 targets use of 36 billion gallons of biofuels per year by 2022. Achieving this may require substantial changes to current transportation fuel systems for distribution, dispensing, and use in vehicles. The U.S. Department of Energy and the National Renewable Energy Laboratory designed a system dynamics approach to help focus government action by determining what supply chain changes would have the greatest potential to accelerate biofuels deployment. The National Renewable Energy Laboratory developed the Biomass Scenario Model, a system dynamics model which represents the primary system effects and dependencies in the biomass-to-biofuels supply chain. The model provides a framework for developing scenarios and conducting biofuels policy analysis. This paper focuses on the downstream portion of the supply chain–represented in the distribution logistics, dispensing station, and fuel utilization, and vehicle modules of the Biomass Scenario Model. This model initially focused on ethanol, but has since been expanded to include other biofuels. Some portions of this system are represented dynamically with major interactions and feedbacks, especially those related to a dispensing station owner’s decision whether to offer ethanol fuel and a consumer’s choice whether to purchase that fuel. Other portions of the system are modeled with little or no dynamics; the vehicle choices of consumers are represented as discrete scenarios. This paper explores conditions needed to sustain an ethanol fuel market and identifies implications of these findings for program and policy goals. A large, economically sustainable ethanol fuel market (or other biofuel market) requires low end-user fuel price relative to gasoline and sufficient producer payment, which are difficult to achieve simultaneously. Other requirements (different for ethanol vs. other biofuel markets) include the need for infrastructure for distribution and dispensing and widespread use of high ethanol blends in flexible-fuel vehicles.

## Introduction

Transportation fuels from biomass (biofuels) are pursued to achieve multiple goals: reduced petroleum use and greenhouse gas emissions, fuel oxygenation, and agricultural market diversification [1], [2]. This paper focuses on ethanol. Where real-world systems or modeled system representation is likely generalizable across all biofuels, we use the term “biofuel” to be broadly inclusive of all such fuels, including ethanol. In some cases we refer to “ethanol or other biofuel” to highlight that this analysis emphasizes ethanol but the statement in question applies broadly as well. We refer to “ethanol” for items that apply to that fuel only, and we refer to “ethanol” in presenting all results of the analysis because they are for ethanol specifically.

The U.S. government has a long-standing goal of reduced dependence on imported petroleum that was initially prompted by the oil crisis and embargo of 1973. Federal incentives for the production and use of biofuels have been enacted as one method to achieve that goal. The Clean Air Act Amendments of 1990 (Public Law 101–549, 42 U.S.C. 7401) and associated fuel regulations established oxygenation requirements for much of the nation’s gasoline supply, which oxygenated biofuels such as ethanol can help meet; these were subsequently supplemented with renewable fuel requirements [3], [4]. More recently, the federal government has taken judicial, regulatory, and legislative steps to reduce greenhouse gas emissions. On April 2, 2007, the Supreme Court found in *Massachusetts v. EPA*, 549 U.S. 497 that greenhouse gases are air pollutants, resulting in regulatory action to limit greenhouse gas emissions from motor vehicles [5]. Climate change legislation has been introduced in Congress [6]. Low-carbon biofuels can help meet greenhouse gas mitigation goals.

In acknowledgment of the potential contribution of biofuels to achieving these and other goals, recent policies at the state and federal levels in the United States have promoted the use of biofuels, with a long-term focus on ethanol and other biofuels. Such policies include:

- a federal biofuels research, development, and deployment program
- preferential tax treatment of biofuels production and sales
- subsidies for investments in fueling infrastructure
- flexible-fuel vehicle (FFV) value for corporate average fuel economy compliance
- renewable fuels standards [3], [4], [7].

Despite this history of policy intervention, biofuels use in the transportation fuel market remains small (10.7 billion gallons [8]) compared with the 36 billion gallons of biofuels by 2022 specified in the Energy Independence and Security Act of 2007 (EISA). Challenges to increased use include retail gasoline prices that are lower than the cost of delivering biofuel to the pump, logistics and infrastructure requirements for fuel distribution and dispensing, biomass-to-biofuel conversion cost and capital investment requirements, and logistical and market issues of biomass supply. Many of these challenges arise because the supply chain for biofuels differs substantially from the supply chain for petroleum-based transportation fuels [3], [7]. Infrastructure-compatible biofuels (non-ethanol) would mitigate some, but not eliminate all, of these differences.

**Figure 1:**
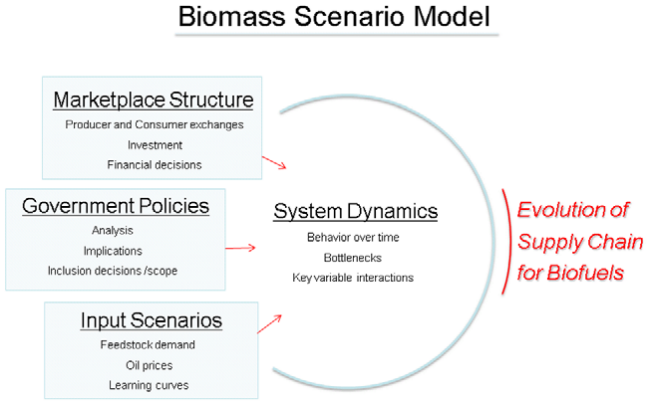
Overview of Biomass Scenario Model. The figure shows the overall purpose and content of the Biomass Scenario Model.

To analyze such challenges, the U.S. Department of Energy (DOE) and the National Renewable Energy Laboratory developed a system dynamics modeling approach that represents the primary system effects and dependencies in the biomass-to-biofuels supply chain [9], [10]. For purposes of this analysis, the biomass-to-biofuels supply chain is discussed at the overall industry level and with a focus on development and evolution of a supply chain for a developing industry. This is not to be confused with the day-to-day management of the existing supply chain of an individual firm working with other individual firms. In this context, the dynamics in question relate to the development of entire sectors of the industry on a year-to-year timescale (e.g. how long does it take production capacity to develop), not to supply chain management dynamics of individual firms that play out over weeks, days, or even hours (e.g. how long does it take this part to be shipped). This approach was designed to support biofuels policy analysis by determining what supply chain changes have the greatest potential to accelerate the deployment of biofuels (see Figure 1). In this paper, we address the “downstream” portion of the supply chain, including the distribution logistics, dispensing station, fuel utilization, and vehicle portions of the chain. The DOE-sponsored system dynamics model of the biofuels supply chain–the Biomass Scenario Model–represents major interactions and feedbacks related to a dispensing station owner’s decision to offer ethanol fuel, with distribution options and vehicle choice represented as discrete scenarios.

**Figure 2:**
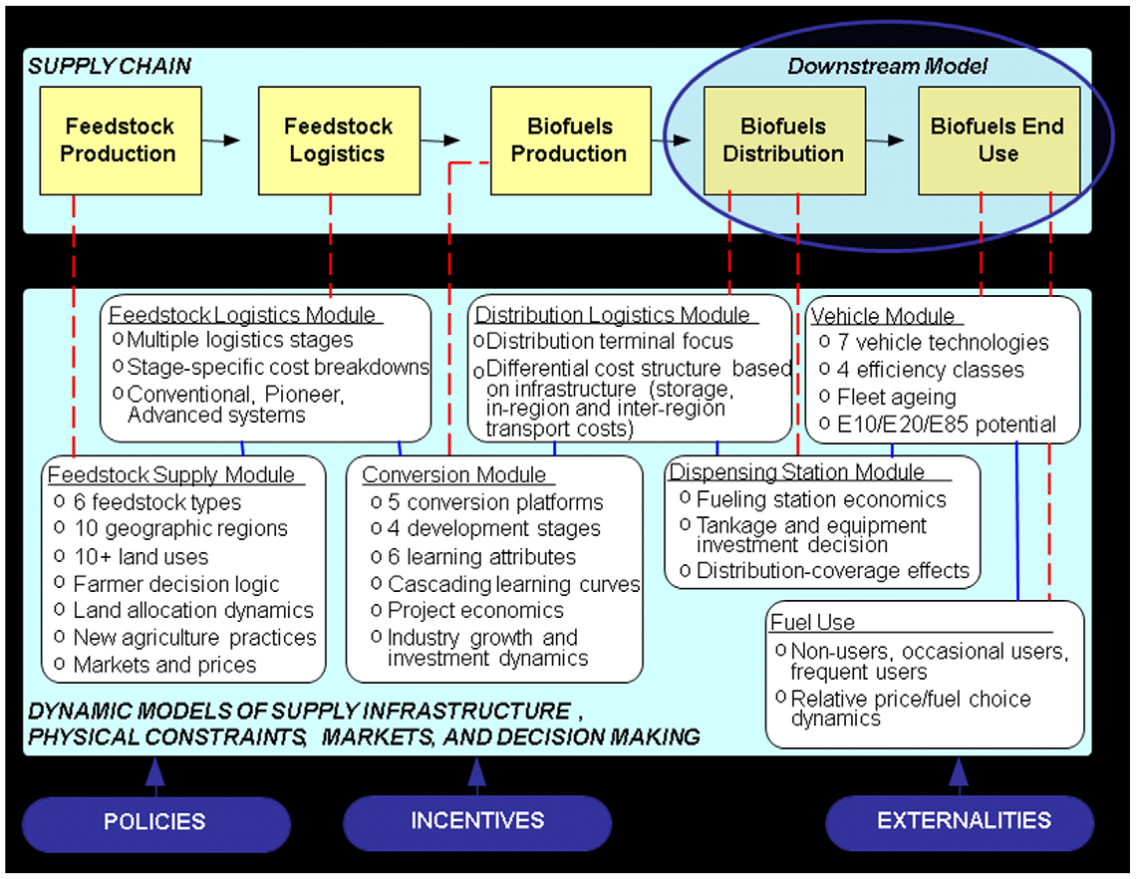
Supply chain for ethanol fuel with Biomass Scenario Model structure. The full supply chain for ethanol fuel is shown, with the downstream end labeled. The modules of the Biomass Scenario Model are briefly described. The downstream version of the Biomass Scenario Model that was used in this paper includes a simplified version of the Conversion Module and the Distribution Logistics, Dispensing Station, and Vehicle Modules. For simplicity, in the downstream version, the Vehicle Module is used to generate vehicle scenarios, rather than being fully coupled during each run. E10, E20, and E85 are light-duty vehicle fuels that are approximately 10%, 20%, and 85% ethanol by volume, respectively.

This paper addresses the downstream section of the supply chain. The full supply chain is shown in Figure 2; the downstream end of the chain elaborates on the biofuels distribution and end-use components that are noted in the figure. Characteristics of the major components of the downstream system are summarized in Table 1. For each linked element in the downstream supply chain (i.e., entries in the first column), the infrastructure and market conditions necessary for an economically sustainable industry are listed, along with policies that can shape these infrastructure and market conditions. In the Methods section, we describe each of the supply chain links, infrastructure and market elements, and policy levers, as well as their representation in the model.

In considering the downstream portion of the biofuels supply chain, three major analytic questions guide our exploration:

1. Can an economically sustainable domestic ethanol fuel distribution, dispensing, and end-use system develop, and under what policy and market conditions?
2. What combination of policies (including carbon policy) appears most likely to achieve policy goals?
3. How do these conclusions differ under different sets of assumptions about consumer and system behavior?

**Table 1:** Infrastructure and Market Needs and Related Policies across the Downstream Supply Chain.

Supply Chain Step	Infrastructure	Infrastructure Policies	Market Conditions	Market Policies
Conversion	Conversion capacity	None	Return on conversion investment	Point-of-productionsubsidy
Distribution	Tankage, terminal, truck, pipeline, rail capacity	Distribution and storage subsidy	Return on distribution investment	None
Dispensing	High-blend tankage and dispensing equipment	Repurposing subsidy; fixed capital investment subsidy	Return on investment in tankage and equipment	None
End Use	Flexible-fuel vehicles	Manufacturer incentives; Car Allowance Rebate System (“Cash for Clunkers”) or other consumer incentives for vehicle purchase	Low relative end-use fuel price	Point-of-use subsidy; incremental gasolinecost

The table shows supply chain steps, infrastructure and infrastructure policies at each step, market conditions needed for ethanol market growth or stability, and market policies that apply at each step.

We found that an economically sustainable domestic ethanol fuel distribution, dispensing, and end-use system could develop under conditions of strong policy intervention or significant change from current market conditions. Results suggest that, with sufficient policy intervention (or sufficient market change) across the supply chain, the policy goals could be achieved. However, policy intervention or market change relative to today’s conditions would be necessary: competitive prices at the pump to attract consumers, financially adequate revenue streams to attract producers, and the infrastructure to bring the fuel to market are all necessary elements for a sustainable market, and do not exist at this time, nor are they envisioned in a business-as-usual situation. When assumptions about consumer behavior and system behavior differ, these conclusions generally persist, although substantially different assumptions affect the level of investment in incentives that would be required to develop and sustain an ethanol fuel system.

The remaining sections of the paper will explore these questions, and possible answers, based on modeling results.

## Methods

This Methods section describes each part of the biomass supply chain and briefly identifies what modules of the Biomass Scenario Model represent each part. The sections below offer more detail on how each part of the supply chain is represented. The Biomass Scenario Model is an analytic model that uses a system dynamics approach [11]. It is built in STELLA software [12]. The full model represents dynamic interactions in the biomass-to-biofuels supply chain, including the five interdependent systems shown in Figure 2: feedstock production, feedstock logistics, biofuels production, biofuels distribution, and biofuels end use [13]. This analysis uses a portion of the full Biomass Scenario Model, the downstream model. An analysis that uses the full model is underway and will explore the broader context for the downstream system addressed in this paper. The model is not intended to forecast the future; high-level models (such as the Biomass Scenario Model) are imprecise and best suited for analysis that focuses on relationships, interactions, and trends rather than on single-point predictions. Both the model development process and its results can help identify suspect assumptions and can thereby suggest agendas for further investigation.

The downstream portion of the biofuels supply chain, and the corresponding downstream portion of the Biomass Scenario Model, encompasses a complex physical and market system that transfers biofuel from biomass-to-biofuel conversion facilities to its point of use as a transportation fuel. Table 2 shows selected assumptions that are used in this analysis in the downstream model.

**Table 2:** Selected Assumptions.

Item and Description	Value	Notes
Maximum cellulosic feedstock production and corresponding ceiling for cellulosic feedstockprice	500 million tons/yr;$105/ton	The feedstock production is an input assumption based on EISA 2007. Theceiling price is an input consistent with that production level.
Initial starch ethanol production capacity (Jan. 2006)	5×10^9^ gal/yr	During simulation, production varies in response to price.
Maximum starch ethanol production capacity	15 billion gal/yr	Constraint on starch ethanol eligible for federal Renewable Fuel Standardunder EISA 2007.
Maximum cellulosic ethanol production capacity	44 billion gal/yr	Based on feedstock production input assumption and an assumed 90 gal/tonconversion rate (500 million tons/yr×90 gal/ton).
Maximum ethanol imports	1×10^9^ gal/yr	This ceiling is consistent with imports during the period 2007–2009 whenimports reached this level (but then collapsed).
Initial ethanol point-of-production price (Jan. 2006)	$1.50/gal	During simulation, price varies to equilibrate supply and demand.
Minimum point-of-production price for cellulosiccapacity addition	$1.20/gal $2.40/gal	For fully developed cellulosic industry. For initial-condition cellulosic industry.
Minimum point-of-production price for normalcellulosic industry utilization	$1.00/gal $2.00/gal	For fully developed cellulosic industry. For initial-condition cellulosic industry.
Distribution and storage cost	$0.25/gal $0.05/gal	Input assumption for intra-region, no infrastructure. Input assumption forintra-region, with infrastructure.
Cost of moving ethanol from one region to another	$0.10/gal	Input assumption for movement between any two regions.
Transportation cost from distribution terminal to retail station	$0.04/gal	Input assumption for point-of-distribution to point-of-use delivery cost.
Cost per station of repurposing existing tankageand equipment	$20,000	Existing mid-grade equipment repurposed for high-blend fuel storageand dispensing.
Cost per station of new tankage and equipment	$60,000	Purchase and installation of new equipment for high-blend fuel storageand dispensing.

The table shows some of the major assumptions that are used in the model.

Biomass conversion to biofuel, which occurs at the upstream end of the chain, is beyond the scope of our analysis in this report. In order to depict the co-evolution of the downstream and conversion systems, we developed a simplified conversion module in the downstream model. Conversion is represented in greater detail in the full Biomass Scenario Model.

**Figure 3:**
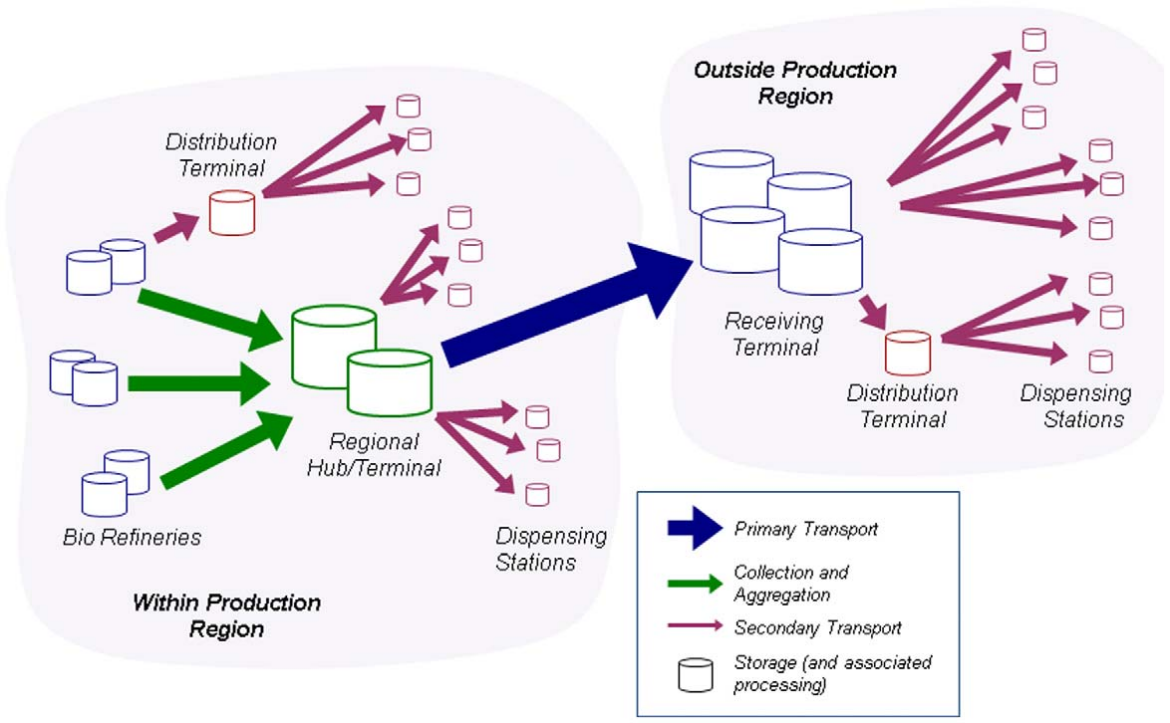
Ethanol distribution system. This conceptual view depicts the path of ethanol transport from biorefinery to dispensing station–the ethanol distribution system. Storage capacity is included in the figure. Separation between ethanol and gasoline is generally maintained until the distribution terminal. The distribution logistics module of the Biomass Scenario Model represents the distribution system and represents expansion of that system due to supply push–in which supply of ethanol coming out of the biorefineries that is in excess of distribution capacity prompts greater distribution capacity growth. Distribution within the production region (intra-region distribution) and from one production region to another (inter-region distribution) is shown.

Increasing the availability of biofuels within the distribution system is a challenge. In the case of ethanol it must be kept separate from gasoline until the distribution terminal because of its chemical properties. At the distribution terminal, ethanol and gasoline are blended, and the blend is transported to a dispensing station (Figure 3).

Truck or rail is generally used to transport ethanol from conversion facilities to distribution terminals, although at least one distributor, Kinder Morgan, uses a gasoline pipeline after careful testing to ensure adequate exclusion of water and oxygen [14]. In many cases, the current pipeline network is not appropriately located for ethanol or biofuel distribution. The likely regional distribution of domestic biofuel production is quite different from the regional population distribution and associated demand for transportation fuel, which exacerbates the challenge of developing a biofuel distribution system. In the Biomass Scenario Model, the distribution logistics module represents the distribution system (for more information, see the Distribution Logistics section).

For ethanol or other biofuels marketed at retail, station owners must decide to offer the biofuel if it is to become more widely available at the pump. High-blend fuel is a blend of ethanol and gasoline with a high percentage of ethanol. An example of a high-blend fuel is E85, which targets approximately 85% ethanol content by volume. High-blend fuel requires dedicated tanks, which represent a significant investment for station owners. Station owners also must consider risks of deciding to offer the fuel, such as risk of mishap or loss of sales during construction, liability risks associated with possible misuse of the fuel (i.e., if high-blend ethanol were used in the wrong engines), and environmental contamination (i.e., if ethanol mobilized hazardous chemicals from leaking underground storage tanks). Whether or not the magnitude of these risks is significant, station owner perception of risk is a factor in their decisions. Dispensing stations operate in a very competitive market with thin profit margins and substantial shares of revenue from in-store sales. In this context, the choice to dispense biofuel does not lead to clear economic advantage at this time [15]. In the Biomass Scenario Model, the dispensing station module represents the dispensing system.

We adopt an approach to consumer’s choice based on rational choice theory and model price and availability of retail biofuels as determinants of consumer choice. While other factors play a role in choice, consumers are unlikely to seek out biofuels that are more expensive or require extra driving to obtain [15], [16]. Complicating the understanding of price difference is the fact that ethanol has lower volumetric energy content than gasoline, and consumer awareness of this difference varies. The fuel utilization module of the Biomass Scenario Model represents consumer fuel choice.

Major factors in the downstream system include availability of vehicles that can use fuel with high ethanol content; a consumer’s choice to use the fuel; a dispensing station owner’s choice to offer the fuel; and availability of the fuel within the distribution system. These factors would be of little or no significance for infrastructure-compatible biofuels that were blended with regular gasoline and not marketed separately at retail. Such fuels are being addressed in a new version of the Biomass Scenario Model that was not used in this analysis. Federal rules currently allow conventional gasoline vehicles to use fuel with up to 10% ethanol content [17]. EPA has partially granted a waiver that would allow use of gasoline that contains up to 15% ethanol in certain recent model years of vehicles. The current national level of ethanol use for transportation fuel is close to 10%. This national level is called the “blend wall” because this is the maximum amount that can readily be used under current rules. Higher levels of usage would require greater use of high-blend fuel in FFVs, conventional gasoline vehicles certified to a higher ethanol content fuel, biofuels other than ethanol that could be used in conventional gasoline vehicles at shares greater than 10%, or completion of federal rule revisions to allow up to 15% ethanol in gasoline. In the present version of the Biomass Scenario Model, a vehicle module that takes into account FFVs is used to estimate the potential for ethanol use in light-duty vehicles. Different vehicle mixes are explored as scenarios, but the vehicle module is not dynamically linked, and therefore is unresponsive to modeled changes in ethanol price.

In the sections below, we define each element of the downstream supply chain, describe how it is represented in the model (including interactions and limitations), and describe policies that can be applied to each element and how those policies are represented in the model.

### Conversion

Conversion is the transformation of biomass feedstock into biofuels. A full analysis of conversion using the Biomass Scenario Model is underway, but detailed analysis of conversion is not the focus of this study. For this study, we use a highly simplified representation that constrains the growth of conversion capacity while allowing for simple price feedbacks and per-gallon subsidies at the point of production. This dynamic coupling to the downstream modules allows conversion capacity to grow in response to ethanol or other biofuel price and demand. The simplified conversion module represents the acquisition of production capacity for corn and cellulosic ethanol in each of the 10 agricultural regions employed by the Biomass Scenario Model.

There are several important aspects to the logic of this simplified conversion module:

- It represents both corn and a single cellulosic pathway for production of ethanol. (The more detailed conversion module captures multiple cellulosic pathways.)
- A “ceiling” for cellulosic conversion capacity is set as a scenario. This level is consistent with feedstock availability, based on simulations from the feedstock supply module, which is not included in the downstream model.
- Plant financials and investment decisions are captured implicitly using a Bass diffusion formulation [18].
- Feedback from price drives capacity acquisition and utilization.
- The model uses a highly simplified representation of learning curve dynamics, in which the strength of price feedback on industry growth is a function of installed conversion capacity.

Overall, then, the simplified conversion module represents growth trajectories for conversion capacity that are consistent with feedstock availability and responsive to price signals from downstream. Thus, this downstream analysis does not address questions, such as dynamics of changes in cost of production, that would require the detail of the full conversion module.

Despite these limitations, the simplified conversion module permits representation of relationships between ethanol or other biofuel price and both the magnitude and the utilization of conversion capacity. For example, an increase in consumption can drive the price of the biofuel upward. This price signal from the downstream module influences both the acquisition and the utilization of capacity, which in turn changes ethanol production, which feeds back to put a downward pressure on price. Representing this feedback loop–an important market dynamic–is a major purpose of the simplified conversion module. The point-of-use ethanol price in the downstream model is set to cover costs of delivering the fuel, plus an assumed markup (“cost-plus” pricing). An alternative pricing representation would be to assume that if ethanol costs (including subsidies) are below gasoline price, that the ethanol price is set to match the gasoline price (market-based pricing). A new version of the Biomass Scenario Model allows for the user to choose how to represent point-of-use ethanol price, with choices varying from the cost-plus price to the market-based price. In addition to representing this feedback loop, the simplified conversion module allows for dynamic relationships among demand, interregional transport of biofuel, and relative levels of conversion capacity utilization across regions. If demand increases in a region with underutilized conversion capacity, conversion in that region will ramp up and transport of biofuel into that region will decline.

Policies to advance conversion range from research and development to subsidies for conversion facilities. A full analysis of these policies is beyond the scope of this paper on the downstream system. In the downstream version of the model, representation of incentives for conversion is limited to a point-of-production subsidy. The point-of-production subsidy increases the price of ethanol or other biofuel that the conversion sector perceives, but does not directly affect fuel prices further downstream. This increases the incentive for investment in conversion capacity through the mechanisms described above by providing greater returns on conversion investment and by facilitating higher plant utilization rates at a given market price.

Inclusion of this subsidy in the downstream model serves as a proxy for initiatives aimed at building industry capacity through per-gallon subsidies. In the full conversion model, the same price signal is used, but there it enters into an explicit calculation of the conversion facility investment decision that also includes a broader set of subsidies (feedstock, conversion facility output, and investment).

### Distribution Logistics

In our analysis, distribution logistics is the set of organizational capabilities and infrastructure required to move biofuel from conversion facility to dispensing station. Generally, distribution logistics entails two steps: transportation from conversion facility to distribution terminal, and transportation from distribution terminal to dispensing station. The chemical characteristics of ethanol complicate distribution logistics because ethanol generally cannot be moved through pipelines used for petroleum products, stored in the same tanks as petroleum products, or blended with gasoline to make high-blend fuel until it is ready to be transported to the dispensing station. Mitigation of these limits is being explored. For purposes of this analysis, however, we assumed ethanol would require its own infrastructure for these steps and that the availability of high-blend fuels in the distribution system would therefore depend on development of ethanol distribution infrastructure–not just on development of operational and regional conversion capacity [7].

In developing the distribution logistics module, the goal was to explore implications of distribution infrastructure, despite substantial uncertainty as to when, where, and with what component parts the infrastructure might develop. Rather than attempt to model detail about distribution infrastructure expansion, and then explore many scenarios to account for uncertainty, we developed a framework for capturing scenarios around the role of distribution infrastructure within the larger system. These are expressed as (1) different rates of infrastructure development based on evolution of conversion system and (2) cost implications of the evolving distribution infrastructure.

The distribution logistics module categorizes distribution terminals as either possessing or not possessing the infrastructure required to handle ethanol in high volumes. The module provides a framework for estimating the costs of storage and transport of ethanol, both within and across regions. As ethanol infrastructure increases within a region, costs decrease for “importing” ethanol from other regions, for storing ethanol within the region, and for transporting ethanol within the region. The module is structured such that additional ethanol supplies in a given region will increase ethanol infrastructure penetration within that region first, and the additional supply will then encourage ethanol infrastructure additions in other regions (see Figure 3). This simplifying assumption is an imperfect representation of market behavior and could misrepresent regional flows when regional ethanol prices make other patterns of distribution more profitable. However, a more realistic representation was deemed overly complex for this version of the model. The distribution logistics module incorporates “supply push” pressure from the upstream side that could cause distribution terminals to add ethanol infrastructure capability.

We do not represent the economics around the infrastructure investment decision. Instead, we simply assume that supply push pressure drives the acquisition of infrastructure over time. This rate of infrastructure acquisition is not instantaneous for a wide range of financial and physical reasons. In the model, the assumed rate of infrastructure acquisition can be changed to approximate the effect of more or less favorable financial conditions for investment, but any particular rate is not explicitly related to financial assumption. The point-of-distribution subsidy can be used to vary the constraints imposed by distribution infrastructure. The 10-region structure of the Biomass Scenario Model permits exploration of broad regional differences in ethanol distribution, but does not permit exploration of distribution logistics below the regional level. The model also represents ethanol or biofuel imports, which are being explored in other analyses.

The primary interaction of the distribution logistics module with upstream modules is the supply push mechanism by which excess ethanol supplies motivate the acquisition of ethanol infrastructure at distribution terminals. The modules farther downstream do not directly feed price signals back to the distribution logistics module; instead, those price signals influence production, which in turn determines the amount of supply push. Because terminals that acquire ethanol infrastructure are assumed to retain it, no feedback is represented in the model that would cause terminals to stop offering ethanol once they had started. The distribution logistics module inputs to downstream modules by way of three variables: the availability of ethanol within the distribution system, a factor in the dispensing station owner’s decision to offer high-blend fuel, and the cost of distribution, which is a component of fuel price at point of use.

Policies that encourage investment in distribution infrastructure could provide incentives that may be essential for its development. In the downstream model, these policies are represented by a per-gallon subsidy to storage within the distribution system–the distribution and storage subsidy–and this lower cost is passed down the supply chain, ultimately reducing point-of-use price to consumers and possibly increasing demand.

### Dispensing Stations

Approximately 120,000 dispensing stations (as of 2007) provide ready access to vehicle fuel in most parts of the United States [19]. Dispensing stations obtain fuel by truck from the distribution network, store it in underground tanks, and pump it into consumers’ vehicles. Stations generally offer at least two grades of gasoline. In addition, a third, mid-grade gasoline, as well as diesel fuel, are widely available, and some dispensing stations offer other alternative fuels (for more information on alternative fuel availability, see http://www.afdc.energy.gov/afdc/locator/stations/).

Dispensing high-blend fuel requires significant investment at the dispensing station, including costs related to fuel storage tanks, pumps, management of legal and liability issues, and customer communications. Of these, the tankage investment is the most significant up-front issue, and stations can be categorized by the type of tankage investment needed. Some stations may have three different types of fuel tanks for gasoline–one for each grade. Because pumps can blend low- and high-grade fuels to create mid-grade fuels, the mid-grade tank at these stations can be repurposed for high-blend ethanol fuel. A station that does not have a mid-grade tank that it could repurpose would have to install a new tank for high-blend fuel. Installing a new tank requires greater investment than repurposing one [15]. Costs to obtain dispensing infrastructure at a station could range from $2,500 at the lowest (for repurposing) to $200,000 at the highest (for installing new equipment in a high-cost location). Table 2 shows cost assumptions used in the model. Dispensing high-blend fuel offers station owners little certainty of increased profit: profit on fuel sales is low relative to profits on in-store inventory; increased sales of high-blend fuel represent reduced sales of gasoline (overall, though not necessarily for each station); and the effect of high-blend fuel availability on in-store sales is uncertain.

The dispensing station module represents, in considerable detail, the station owner’s choice to invest in high-blend ethanol dispensing equipment. Because the model does not include vehicle choice, this decision is not part of a dynamic interaction of consumer vehicle choice, fuel availability, and distribution capacity. Given that FFV owners are often unaware of their vehicles’ flex-fueling capability and that FFV owners now refuel with high-blend only 4% of the time [8], this interaction is unlikely to be strong in the near term. The module first considers whether an individual station owner has access to ethanol in the distribution network. If so, the module represents the decision to consider installing ethanol-dispensing equipment. The module assumes that specific ownership categories (oil company owned, branded independent, unbranded independent, and hyper-mart stations) face different financial circumstances and market decisions. The module divides stations into those with “repurposable” mid-grade tanks and those without.

A favorable net present value of the investment is a necessary condition for consideration. Net present value is calculated in a detailed pro forma that takes into account the expected net change in revenue and the investment cost associated with the investment in tankage and equipment. The calculation of change in revenue included low-blend sales, high-blend sales, in-store sales, and sales volume for each type of sale. The calculation of investment cost includes investment subsidies for fixed capital investment and repurposing subsidies, interest, taxes, and depreciation, and is discounted at different rates representing changes in required rate of return with change in depreciation or loan status. The point-of-use subsidy improves the net present value by reducing the cost of high-blend.

Other issues, such as competitive considerations, are also represented. The station owner is represented as having perfect knowledge of FFV ownership shares among current and prospective customers–a simplifying assumption. The module explicitly calculates revenues from in-store sales, which account for a substantial share of station revenues and profits. In our base model assumptions, high-blend fuel, because of its lower energy content, results in more frequent fuel stops and slightly higher in-store sales; because this assumption is uncertain, we explore it in a sensitivity analysis (see Behavior and Market Sensitivity Analysis Results).

Despite this detail, data to fully describe the station owner’s decision process are limited, so the analysis incorporates sensitivities on several uncertain parameters. Other significant data limitations include inputs for tankage cost estimates. Cost is modeled as depending only on whether repurposing occurs or a new tank is installed, whereas actual installed costs of high-blend tankage are likely to depend significantly on other factors, such as whether station configuration makes tank repurposing or new purchase easy or difficult.

The dispensing station module, like the distribution logistics module, interacts with other modules both by way of availability of high-blend fuel within the distribution system and via price. Greater availability of high-blend fuel dispensing capacity means that, in the fuel utilization module, more FFV owners have the option to choose high-blend. Retail pricing at the point-of-use is calculated as the cost of delivering ethanol or other biofuel to the dispensing station plus an assumed markup (“cost-plus” pricing). An alternative pricing representation would be to assume that if biofuel costs (including subsidies) are below gasoline price, that the biofuel price is set to match the gasoline price (market-based pricing). A new version of the Biomass Scenario Model allows for the user to choose how to represent point-of-use biofuel price, with choices varying from the cost-plus price to the market-based price. The model seeks an equilibrium between production and consumption of biofuel in the entire supply chain without explicit representation of a retail-level market. If retail demand increases, lower inventory increases upstream price, ultimately feeding back to retail price.

Because the station owners’ investment decision is represented in more detail than conversion and distribution investments, financial incentives for station owners can be represented in more detail. Policy levers within the dispensing station module include a subsidy for investment in new tanks (fixed capital investment subsidy), a tank-repurposing subsidy (repurposing subsidy), and a per-gallon subsidy to the price of fuel at point of use (point-of-use subsidy). These modeled incentives mirror the types of incentives that have been used to encourage industry growth.

### Fuel Utilization and Vehicles

Combustion of ethanol or gasoline in light-duty vehicles is the fuel utilization considered here, and other vehicles and fuels are not considered. Low-blend ethanol fuel and high-blend ethanol fuel involve different decision makers. For low-blend fuel, utilization depends primarily upon the requirements for renewable fuels and limits on low-blend ethanol content (10% by volume is the current legal limit in the United States). The amount of ethanol used if all gasoline were 10% ethanol by volume is referred to as the “blend wall.” EPA estimates that the blend wall will be reached in 2014; the exact timing depends upon the rate of increase in ethanol use, the rate of decrease in gasoline use due to the increasing fuel economy of the light-duty vehicle fleet, and whether or not the legal limit for ethanol content is increased (e.g., to 15%). The blend wall does not apply to infrastructure-compatible biofuels. In contrast to pervasive use of low-blend, utilization of high-blend fuel involves greater complexity, as it requires the availability of vehicles that can burn the fuel, the availability of fuel at the dispensing station, and consumers’ decisions to purchase the fuel. Current law allows high-blend E85 only in FFVs in the United States. Consumer fuel purchase decisions appear to be heavily influenced by, but not entirely dictated by, the price of high-blend compared with gasoline.

The model estimates consumer choice of high-blend ethanol fuel. This estimate is made in the fuel utilization module of the downstream model. This module receives two key inputs from the dispensing station module: a vehicle scenario that determines the share of high-blend-capable vehicles and the availability of high-blend fuel at the dispensing station. It accounts for the regulatory requirements encouraging use of low-blend fuel and estimates consumer choice of high-blend. For FFV owners with access to high-blend dispensing, the fuel utilization module models consumer choice between high-blend and gasoline. Although additional refinements might be more realistic, the module now represents consumer decisions based on the price per unit energy of fuel–not volume of fuel–and it assumes consumers have complete knowledge of availability of high-blend fuel at dispensing stations. Consumers are simplified into two categories: If gasoline and high-blend fuel were identically priced on an energy basis and available, *regular users* would choose high-blend fuel 80% of the time, while *occasional users* would choose gasoline 80% of the time.

The Biomass Scenario Model does have a vehicle module, although it is not dynamically coupled to the downstream model for this study. The vehicle module does not incorporate consumer vehicle choice but instead provides an accounting framework in which to examine potential policy influences on vehicle stocks and associated maximum potential ethanol utilization. The vehicle module can generate policy scenarios that can be used in the downstream model. The pattern over time of maximum ethanol consumption potential under each scenario is an input to the integrated downstream model.

Accordingly, the model in its current form does not represent vehicle choice and related interactions and is limited in its representation of consumer choice of fuel. It does not represent dynamic interactions of fuel price and fuel availability with vehicle choice, nor does it estimate effects of education and outreach efforts on vehicle choice. Dynamic interactions could become a more important consideration if FFVs were refueled with high-blend a greater share of the time and if consumer choice played a greater role in FFV purchase. The model also does not examine consumer fuel choice among atypical consumers (e.g., those who would pay a substantial premium or drive far out of their way to obtain high-blend fuel or those who would decline high-blend fuel under any circumstance). Within the current model structure, because data on consumer fuel choice are limited, we conducted sensitivity analysis on the assumptions about choices of regular users and occasional users (see Behavior and Market Sensitivity Analysis Results).

Policies related to fuel utilization include any policy that lowers the price of ethanol at the pump relative to the price of gasoline and to upstream ethanol prices. Such policies could include carbon taxes or cap policies, gasoline taxes or floor prices, ethanol subsidies or ceiling prices, or other incentives. In the downstream model, these can be represented as gasoline taxes or high-blend fuel subsidies. The increase in cost of high-blend fuel if a carbon tax or a carbon cap policy were implemented would likely be smaller than the increase in the cost of gasoline, although both fuels emit carbon. The downstream model also includes a point-of-use subsidy, which reduces ethanol price at the pump. Vehicle policies–such as the Car Allowance Rebate System (Cash for Clunkers), vehicle purchase incentives, FFV standards, and efficiency standards–can be represented as different maximum potential ethanol consumption scenarios in the downstream model, based on outputs from the vehicle module.

### Structural Summary of Downstream System

The downstream system exhibits the challenge of maintaining a stable, competitive ethanol fuel market. Gasoline is the dominant fuel in the light-duty vehicle market, and the cost of driving a gasoline-fueled vehicle sets a ceiling above which alternative vehicle-fuel systems are unlikely to compete for substantial market share. On the supply side, ethanol at point of production must sell at a price sufficient to compensate upstream market actors–agribusiness, feedstock logistics operators, and ethanol conversion facilities–for their investments and labor. Overall, the behavior of the downstream system reflects the complex feedback between supply and demand, which connect producers and consumers at each stage of the supply chain.

The downstream system also demonstrates the need for coordinated growth across the entire downstream supply chain. Insufficient capacity at any link will inhibit growth of the market overall, as indicated in the results in the Results and Discussion. Different parts of the system respond to signals for investment with different lag times, complicating the necessary coordination in growth across the system.

## Results and Discussion

We used the Biomass Scenario Model to explore how incentives for market actors across the supply chain shape the potential for a sustainable ethanol market in the United States. In this section, we present the results of model runs of the downstream system that were designed to explore these issues. A supporting information file offers a data supplement (Data S1) that includes results of all of the model runs that are presented here, as well as additional model runs.

**Figure 4:**
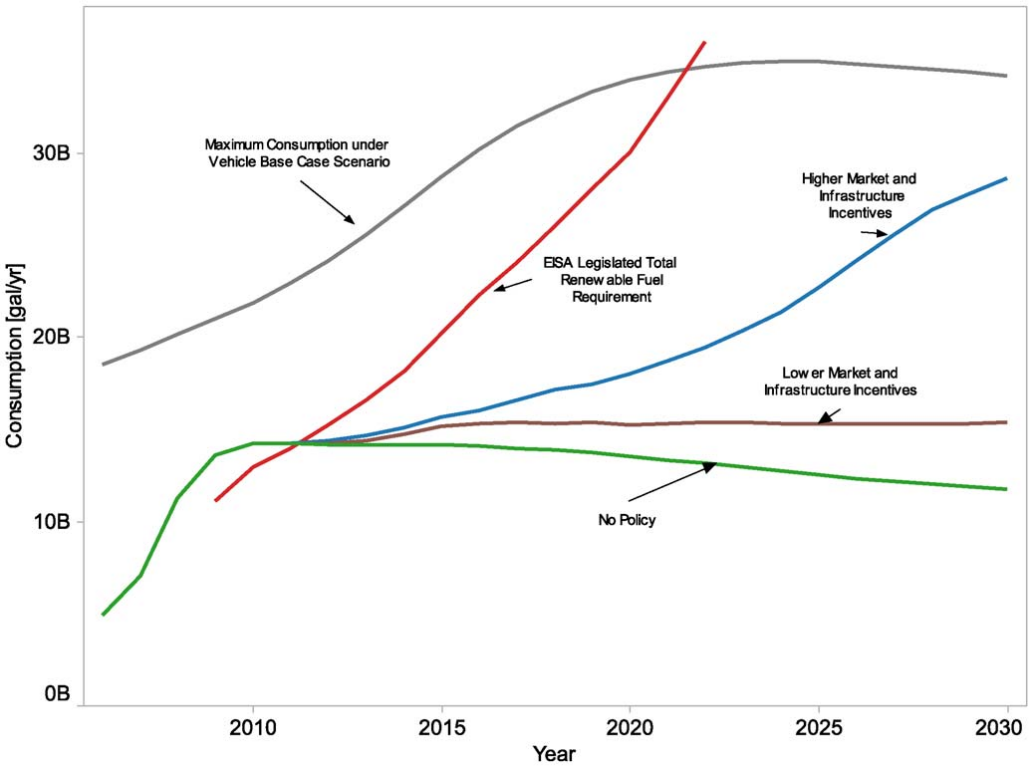
Scenarios for actual and potential ethanol consumption. The figure shows ethanol consumption results for all years from three modeled scenarios: a No Policy case (i.e., no incentives), a Higher Market and Infrastructure Incentive case, and a Lower Market and Infrastructure Incentive case. For comparison, results for maximum potential consumption ethanol consumption with a particular vehicle scenario are also shown (Maximum Potential Consumption under Vehicle Base Case); the vehicle scenario is based on the Energy Information Administration’s *Annual Energy Outlook* [20]. An external dataset, not representing model results, is also shown for comparison: the EISA Legislated Total Renewable Fuel Requirement, which shows the goals of Energy Independence and Security Act of 2007 for renewable fuel (not necessarily ethanol). The figure illustrates that model results suggest that reaching EISA targets would require considerable incentives.

The results displayed in Figure 4 show simulated actual ethanol consumption from several modeled cases: a “No Policy” case without incentives (not even current incentives), a “Higher Market and Infrastructure” case with higher levels of incentives, both on a per-gallon basis and for infrastructure investments, and a “Lower Market and Infrastructure” case with lower levels for these incentives. Incentive levels for these cases are shown in Table 3. Note that none of these cases is intended to represent continuation of current policy. We recognize that the levels of policy intervention explored in some of these cases are significant; this paper does not advocate adoption of these particular policies, nor do we assert that the results presented here are accurate projections of the cost and effect of these policies. Instead, we intend to explore the behavior of the system as modeled, seeking insights on system behavior, not to predict specific numerical results.

**Table 3:** Summary of Incentives.

Policy Category	Incentive Name	Range of Values Considered	Definition and Notes
Market Incentives	Point-of-production subsidy	$0/gal–$1/gal	For cellulosic ethanol producers only.
	Point-of-use subsidy	$0/gal–$1/gal	Lowers ethanol price at point of use. Fiscal effect increases with increasing ethanol use.
	Incremental gasoline cost	$0/gal–$2/gal	Represents any policy or market condition that differentially increases gasoline price at point of use (e.g., carbon policy, high oil price with little ethanol price response). As a tax, the fiscal effect decreases with increasing ethanol use.
Infrastructure Incentives	Distribution and storagesubsidy	$0/gal–$1/gal	Subsidy for tankage and equipment at distribution terminals and for distribution.
	Repurposing subsidy	0%–75%	Percentage of total cost that is subsidized. Subsidy for repurposing of existing dispensing station infrastructure.
	Fixed capital investmentsubsidy	0%–75%	Percentage of total cost that is subsidized. Subsidy for installation of new dispensing station infrastructure.

The table summarizes the market and infrastructure incentives, the range of values for the incentives that are considered in the analysis, and further details about each incentive.

Also shown in the figure, for comparison, is an external dataset that does not represent model results: The “EISA Legislated Total Renewable Fuel Requirement” represents a growth trajectory that would rise to the 36 billion gallons per year of biofuels utilization by 2022 that is targeted in the legislation. The EPA Administrator has discretion to reduce the required level if the targets proposed by Congress cannot be met. Congress has not set production targets for years beyond 2022. Ethanol fuel in light-duty vehicle markets is only one of the pathways to meet this total requirement, but the total is offered for reference here because the legislation does not establish a total level of ethanol or a total amount of light-duty vehicle biofuel.

The results shown here indicate that, under the modeled conditions, reaching ethanol consumption, even well below EISA-legislated targets for renewable biofuels, appears to require policy intervention with substantial overall financial implications. The “Maximum Consumption under Vehicle Base Case Scenario” uses the maximum potential ethanol consumption (i.e., ethanol usage if all the flex-fuel vehicles use high-blend fuel exclusively)implied by a vehicle scenario–in this case, the number and type of vehicles in Energy Information Administration’s *Annual Energy Outlook* reference scenario [20]. In Behavior and Market Sensitivity Analysis Results, we explore how vehicle policy scenarios would change this potential consumption of ethanol.

In the No Policy case, the system will neither develop nor sustain a high level of ethanol use: greater infrastructure investment and more favorable market conditions are both needed for a high level of ethanol use to occur. In other words, the No Policy case results in insufficient investment in the distribution and dispensing infrastructure to support widespread high-blend ethanol use, and also represents a market situation in which high-blend ethanol is not competitive with gasoline fuel. The No Policy case is defined by a set of inputs and a set of assumptions internal to the model. Because the vehicle module is not dynamically coupled to the downstream model, a vehicle scenario that specifies number of vehicles, type of vehicles, and vehicle-miles of travel is part of the input data. The amount of ethanol consumption declines in the later years of the No Policy case because the vehicle scenario assumes that increasing efficiency of the light-duty vehicle fleet reduces low-blend ethanol consumption over time.

The substantial gap that emerges in later years between modeled ethanol consumption, even in the Higher Market and Infrastructure policy case, and the Maximum Consumption under Vehicle Base Case Scenario reflects the numerous challenges that impede penetration of high-blend ethanol fuel in the market. These challenges may include resource and conversion costs that result in uncompetitive pricing for high-blend ethanol fuel, investment cost barriers leading to insufficient investment in distribution and dispensing infrastructure for high-blend fuel, and limitations on sales of high-blend fuel due to low market share of FFVs or low rates of consumer purchase of high-blend fuel. Our results suggest that these challenges might most effectively be addressed through coordinated policy incentives across the supply chain, and we explored market, infrastructure, and vehicle incentives that might close this gap. High-blend-capable vehicles will not always be fueled with high-blend; therefore, additional high-blend-capable vehicles might be needed to approach the legislative target, even for years when the Maximum Consumption under Vehicle Base Case exceeds that target. To ensure that this gap more likely is a characteristic of the system than a problem with the model, we performed a number of tests on the model that demonstrated that the gap can be closed if assumptions about these challenges are altered.

### Policy Analysis Results

The policy analysis results explore different policy design choices, such as which incentives to apply, at what level, with what revenue implications, and over what time period. The results suggest that each section of the downstream supply chain contributes to a functional whole, and so effective policy design takes these interdependencies into account. To explore policy design choices, we altered model inputs to represent effects of different policies, without changing other inputs or calculations. The policies are listed and defined in Table 3. The incentive levels in the policy cases, each set at particular monetary values, are summarized in Table 4. Some of these incentives would require governmental subsidies, while others, notably the incremental gasoline cost, could potentially be achieved through market conditions (e.g., high oil price and breakthroughs in ethanol production could result in higher incremental gasoline cost than today). These incentives apply to different actors at different points in the supply chain. We selected a range of incentive values to model, to explore changes in system behavior under different conditions. This does not imply endorsement of these incentive values. We acknowledge that there will be a diversity of opinion as to whether these incentive levels are politically feasible or fiscally prudent, and we consider this discussion, along with related comparisons of alternate public investments, to be beyond the scope of this paper. The selected monetary values for each incentive become an input to the model and influence the rate of ethanol demand growth and the growth of its supply chain. The results shown here illustrate the magnitude of these influences.

**Table 4:** Incentive Levels in the No Policy and Policy Cases.

Policy Category	Incentive Name	No PolicyLevels	Higher Market andInfrastructure Levels	Lower Market andInfrastructure Levels
Market Incentives	Point-of-production subsidy	$0/gal	$1/gal	$0.50/gal
	Point-of-use subsidy	$0/gal	$1/gal	$0.50/gal
Infrastructure Incentives	Distribution and storage subsidy	$0/gal	$1/gal	$0.50/gal
	Repurposing subsidy	0%	75%	75%
	Fixed capital investment subsidy	0%	75%	75%

The “No Policy Levels” correspond to Run 2804. Runs 3215, 3243, and 3269 have “Higher Market and Infrastructure Incentives” conditions with $0, $0.50, and $1/gal incremental gasoline cost, respectively. Runs 3202, 6901, and 3256 have “Lower Market and Infrastructure Incentives” conditions with $0, $0.50, and $1/gal incremental gasoline cost, respectively.

### Market and Infrastructure Incentive Effects and Costs

Market and infrastructure incentives are two categories of incentives that appear important to designing a policy environment in which ethanol consumption increases. We explore higher and lower levels of market and infrastructure incentives. Market incentives include subsidies at point of use and point of production, as well as an incremental gasoline cost. A larger market incentive could be caused by a variety of policies–a carbon tax, a cap on petroleum, a gasoline tax, or an ethanol price subsidy–or by changed market conditions that keep gasoline prices above ethanol prices. For reference, emission cap levels for greenhouse gases considered in 2009 in the United States would lead to additional maximum cost–if passed through to end users–ranging from $0.24–$0.84/gal of gasoline ($25–$86/ton CO_2_) in 2011 dollars, according to a summary of several different studies (reported in [21] as $0.21–$0.73/gal of gasoline in 2005 dollars. Consumer Price Index calculator (http://data.bls.gov/cgi-bin/cpicalc.pl) and emissions factor from [22] were used for conversions). Effect on cost of ethanol would depend on greenhouse gas emissions rates for ethanol and how these emissions were treated in the policy.

Infrastructure incentives include a distribution and storage subsidy, a repurposing subsidy, and a fixed capital investment subsidy.

We selected the Higher Market and Infrastructure Incentives and the Lower Market and Infrastructure Incentives cases to show a range of additional ethanol consumption over No Policy conditions. We could have selected even higher incentive levels but judged those to be less interesting due to their higher costs. Both the Higher and Lower cases include all policies except an incremental gasoline cost.

Market and infrastructure incentives appear to have a synergistic interaction in the model. Table 5 shows model run results that illustrate effects of different market and infrastructure incentive levels on ethanol consumption (measured in billion gallons per year) in 2022–selected because that year is called out in EISA. Results are shown in columns that indicate whether Market incentives, Infrastructure incentives, or Other incentives (either none or a combination of Market and Infrastructure) are in place. The rows below the “Higher market and infrastructure incentive levels” row explore sensitivities on this case by applying its incentive levels individually and in combination. This isolates the effect of individual incentives. For the selected incentive levels, ethanol consumption does not increase (relative to No Policy) when each market or infrastructure incentive is applied by itself. When all infrastructure incentives are applied together, it increases somewhat, but does not increase when all market incentives are applied (see Table 5). The Higher Market and Infrastructure Incentives case shows synergy–a greater increase in ethanol consumption with both infrastructure and market incentive levels than the additive effect of each set of incentives alone. If the model is a faithful representation of the actual system, this finding would suggest that policy intervention across the supply chain is more effective than a less comprehensive approach.

**Table 5:** Incentive Effect on Ethanol Consumption in 2022.

Name of Case	Ethanol Consumption by Category of Incentive (billion gal/yr)			
	Run	Market	Infrastructure	Other
No policy	2804			13
Lower market and infrastructure incentive levels	3202			15
**Higher market and infrastructure incentive levels**	**3215**			**19**
Point-of-production subsidy	7146	13		
Point-of-use subsidy	7144	13		
**All market incentives at levels in Higher case**	**7152**	**13**		
Repurposing subsidy	2836		13	
Distribution and storage subsidy	7145		13	
Fixed capital investment subsidy	2820		13	
**All infrastructure incentives at levels in Higher case**	**3195**		**15**	

This table summarizes model results for ethanol consumption in 2022. Run refers to model run numbers and can be used to identify corresponding results in the supporting information (Data S1). Runs are grouped into three different categories, shown in columns, depending upon whether Market or Infrastructure incentives are in place. Incentives are applied individually and in combination at “Higher Market and Infrastructure Incentives” levels for the single and multiple policy cases, except for the “Lower Market and Infrastructure Incentives” case which applies all incentives in combination. The “Other” Category includes cases without incentives (No policy), as well as two cases that combine Market and Infrastructure incentives. The rows below the “Higher market and infrastructure incentive levels” row explore sensitivities on this case by applying its incentive levels individually and in combination.

There are different incentive combinations that achieve similar levels of ethanol consumption, indicating significant choice in policy design targeting any particular goal. This is also shown in Table 5, where lower market and infrastructure incentive levels and higher infrastructure incentive levels both reach ethanol consumption of 15 billion gal/year in 2022.

**Figure 5 pone-0035082-g005:**
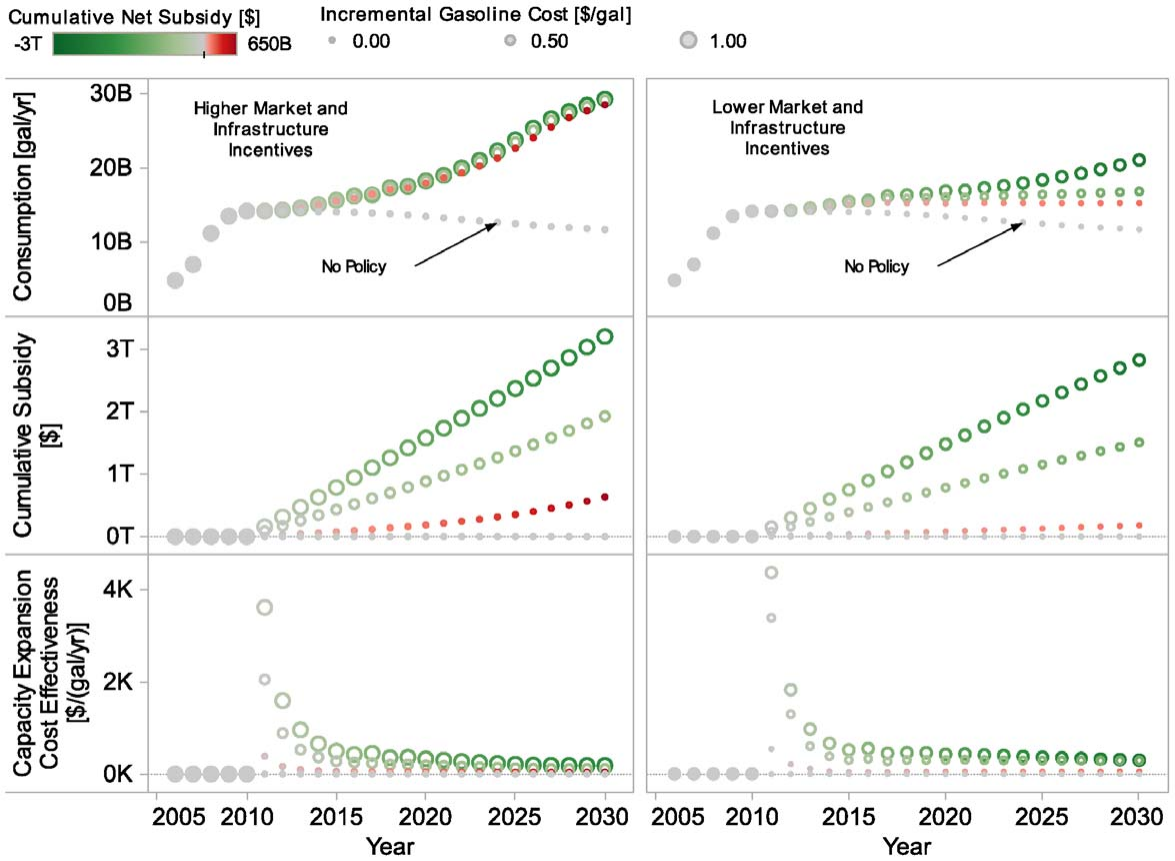
Ethanol consumption, incentive costs, and cost effectiveness with three incremental gasoline costs. The figure shows model results for all years under different policy and incremental gasoline cost conditions. The left-hand column shows results under Higher Market and Infrastructure Incentive conditions, while the right-hand column shows results under Lower Market and Infrastructure Incentive conditions. Three levels of incremental gasoline costs are represented with three different sizes of marks. The cumulative net subsidy color scale shows government payments or revenues generated, assuming that incremental gasoline costs are revenue in the form of gasoline taxes. The top row shows ethanol consumption over time; the middle row shows cumulative subsidy (with incremental gasoline costs treated as a subsidy and added to other costs, rather than being subtracted as they were for the cumulative net subsidy scale); the bottom row shows a metric of effectiveness of investment in annual production capacity–Capacity Expansion Cost Effectiveness.

The effects of a given incremental investment in any single type of incentive depend heavily upon the levels of other incentives and the state of development of the system. This is illustrated in Figure 5, which shows in the top row the effects (on ethanol consumption in billion gallons per year over time) of Higher (left column) and Lower (right column) Market and Infrastructure Incentives at three different levels of incremental gasoline cost. In the top row, four different lines can be observed, corresponding to the No Policy case and $0.00, $0.50, and $1.00 per gallon incremental gasoline cases for each column. With Lower Market and Infrastructure Incentives (right column), changes in the incremental gasoline cost increases ethanol consumption, whereas at Higher Market and Infrastructure Incentive levels (left column), the three lines at different incremental gasoline costs overlap, indicating that little opportunity remains for a $1.00 incremental gasoline cost to have additional impacts on ethanol consumption, because the other incentives embodied in the Higher Market and Infrastructure Incentive set have already increased consumption. The lines with $0.00/gal incremental gasoline cost, appearing as small, light-red dots, correspond to the Higher and Lower Market and Infrastructure Incentives lines in Figure 4, and the 2022 values of these lines for ethanol consumption–19 billion gallons per year (Higher) and 15 billion gallons per year (Lower)–are shown in Table 5.

Higher ethanol consumption levels are reached at significant levels of incentive, and selection of incentive type determines the revenue implications of those incentives. The second row of Figure 5 (Cumulative Subsidy) illustrates the very different revenue implications of different incentive choices. If incremental gasoline costs occurred as a tax, it would generate revenues. This is shown in the figure with green used to indicate positive revenues generated (negative subsidy). The color scale treats incremental gasoline cost as revenue and presents net subsidy as the sum of all incentive costs or revenues. This is why the larger circles, which have higher incremental gasoline costs, are greener. Looking at the left-hand column, the first row shows that similar levels of ethanol consumption are achieved, and the second row shows that this similar level of consumption occurs with three very different cumulative subsidy levels (as seen from the lines) and revenue implications (as seen from the colors). This raises the possibility that combinations of incentives could be selected to yield a desired level of revenue or public cost. Another important contrast between revenue effects of gasoline taxes compared to other policies is the declining need for intervention via gasoline taxes if the ethanol share increases, in contrast to the increasing intervention if ethanol subsidies are used. This is evident in the changes of color over time. These results are not intended to suggest that this level of gasoline tax is desirable, but instead to illustrate the different revenue implications of alternative policy mechanisms for increasing ethanol use.

Cost effectiveness is crucial to successful policy, in particular at the levels of investment that appear necessary to reach a sustained ethanol market. The third row of Figure 5 shows one measure of cost effectiveness. Cumulative costs of policy incentives are divided by incremental annual production capacity relative to the No Policy case, yielding Capacity Expansion Cost-Effectiveness in dollars per gallon-year (higher values imply lower cost effectiveness). This metric should not be confused with the cost of incentives per additional gallon of ethanol. Instead, it is a measure of investment in annual production capacity in the development of the industry. The relative value in different cases, and the trends over time in this metric, suggests greater or lesser cost effectiveness of incentives as an investment in industry transformation. This metric treats incremental gasoline cost as cost, not revenue, to provide a better indication of the overall economic intervention of each package of incentives. This is why Figure 5 shows that cases with higher incremental gasoline costs have higher costs per gallon-year of production achieved. Not surprisingly, the figure illustrates that certain combinations of incentives appear more cost effective than others, and reaching higher levels of ethanol production is associated with use of less cost-effective incentives (higher values).

**Figure 6 pone-0035082-g006:**
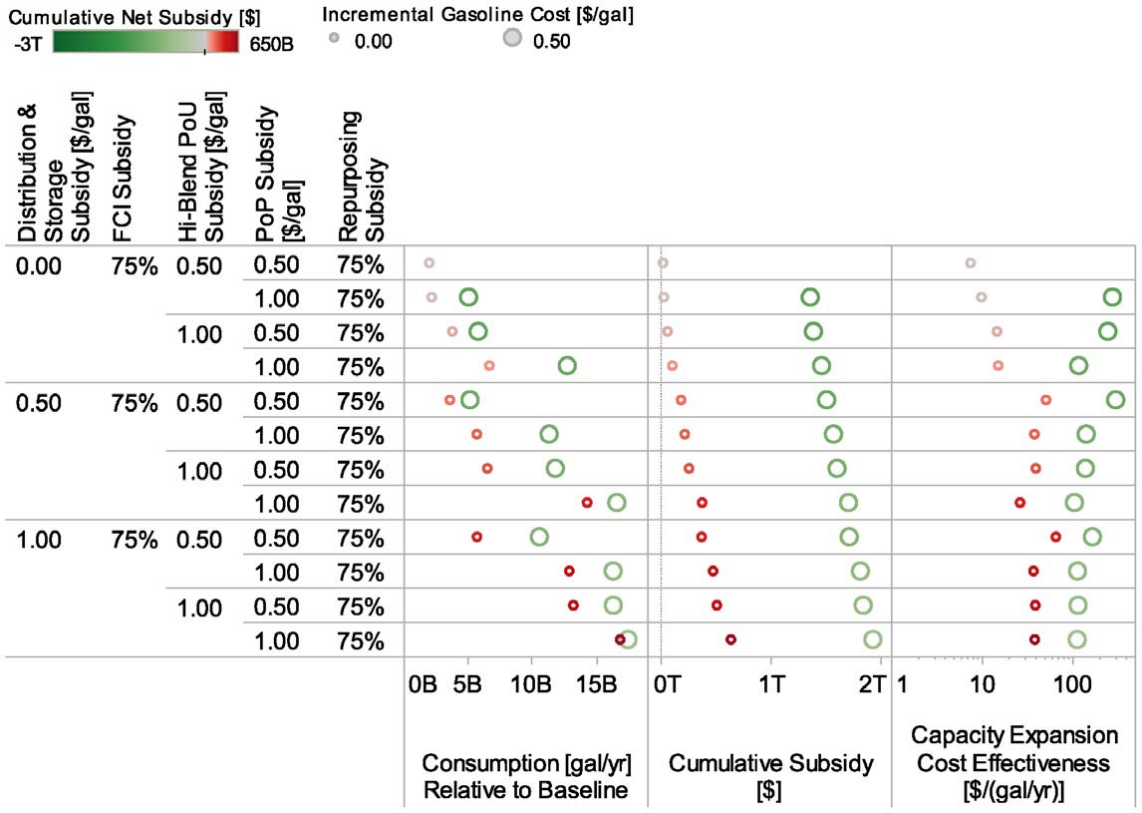
Ethanol consumption, incentive costs, and cost effectiveness of multiple incentive combinations with two incremental gasoline costs in 2030. The figure shows model results for 2030 under different policy and incremental gasoline cost conditions. Two levels for incremental gasoline costs are represented with two different sizes of marks. Policy conditions include three levels of Distribution and Storage Subsidy, one level of Fixed Capital Investment (FCI) Subsidy, two levels of high-blend (Hi-Blend) Point-of-use (PoU) Subsidy, two levels of Point-of-production (PoP) Subsidy, and one level of Repurposing Subsidy. The cumulative net subsidy color scale shows government payments or revenues generated, assuming that incremental gasoline costs are revenue in the form of gasoline taxes. The left-hand column of results shows ethanol consumption over time; the middle column shows cumulative subsidy (with incremental gasoline costs treated as a subsidy and added to other costs, rather than being subtracted as they were for the cumulative net subsidy scale); the right-hand column of results shows a metric of effectiveness of investment in annual production capacity–Capacity Expansion Cost Effectiveness.

The choice of types and levels of incentives, resulting ethanol consumption, and associated costs and cost effectiveness are diverse. Figure 6 illustrates results for a larger set of combinations in a single year, 2030. The three columns of results, like the three rows of Figure 5, show ethanol consumption, incentive costs, and cost effectiveness. As in Figure 5, the cumulative net subsidy color scale shows government payments or revenues generated, assuming that incremental gasoline costs are revenue in the form of gasoline taxes. The green points have $0.50/gal gasoline tax; the red ones have $0.00/gal gasoline tax. The distance between these two points varies for the different rows. This shows how significantly the effect of an incremental $0.50/gal gasoline tax can vary, depending on other incentive levels, indicating once more that effects of a given incremental investment in any single type of incentive depend heavily upon the levels of other incentives and the state of development of the system. Again, the authors do not advocate a particular policy or level of incentive, but explore these scenarios for insights on performance of the system.

These results for market and infrastructure incentives illustrate the importance of a balanced set of incentives, the potential interchangeability among incentives, and the opportunity to select a desired set of incentives based on consumption, revenue, and other considerations. Next, we consider the effects of the duration of incentives.

### Sunset Runs

**Figure 7 pone-0035082-g007:**
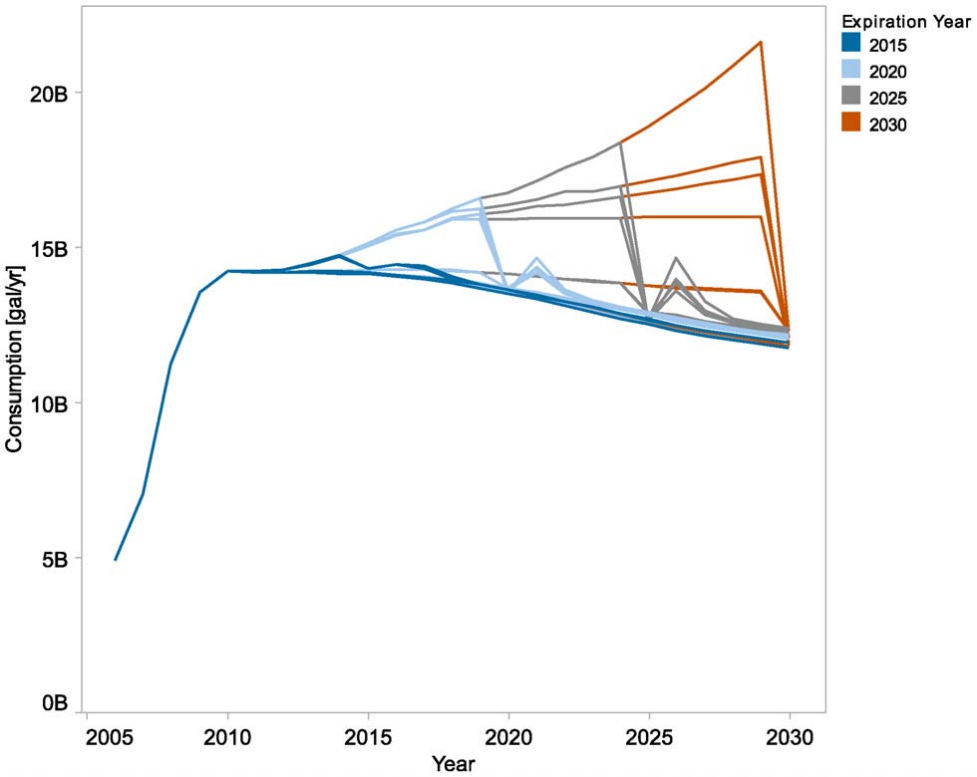
Effect of policy sunset on ethanol consumption. The figure shows ethanol consumption results for all years, with the entire set of policies terminated in the color-coded year. Different lines that have the same policy expiration year represent different sets of policies; however, we did not independently vary the policy duration. The figure shows that the market declines precipitously in the absence of policy intervention, under modeled conditions.

Determining when a policy intervention should terminate is an important element of policy design. In the policy cases, we applied policies throughout the analysis period. Here, we show the results of an analysis of the effect of the timing of policy termination, or sunset. This analysis shows that the model estimates precipitous market decline in the absence of the policy conditions (see Figure 7). This does not imply that the market requires indefinite continuation of policy intervention. To explore the question of what policy intervention portfolio could most effectively establish a self-sustaining market, further analysis would be required of duration, combinations, magnitudes of policy intervention. In this analysis, we did not vary the duration of each policy independently of the other policies or explore the diversity of possible combinations and sequences. For example, we did not terminate infrastructure policies earlier than market policies to assess whether infrastructure policies alone can be phased out after infrastructure development reaches a threshold level, without triggering precipitous market decline. In Figure 7, different lines that have the same policy expiration year represent different combinations of polices. The highest line represents the Lower Market and Infrastructure Incentives case with $2.00/gal incremental gasoline cost, up to the expiration year, while the lines below it have some of the policies turned off during all years. Further analysis would be required to determine whether certain policies could be terminated before others without reducing consumption. Such tests are beyond the scope of this paper but could explore whether infrastructure policies can be terminated once infrastructure penetration reaches a certain share, and after that an end-use price differential alone (whether the result of policy intervention or market conditions) would be sufficient to sustain the market.

In Figure 7, the market appears to collapse the year of the expiration date because there is a single point for each year, and the expiration date year is calculated with the policy absent. The downstream model does not include sufficient detail about the conversion investment decision to estimate the effect of anticipated policy sunset on conversion facility investment decision, so this version of the model effectively assumes that investment decisions are made based on current conditions. More detailed modeling of these decisions would likely cause market decline to start substantially before the sunset year. The oscillations (most visible in the 2020 and 2025 sunset cases) occur because there is ethanol inventory that will be consumed once its price drops.

### Behavior and Market Sensitivity Analysis Results

**Figure 8 pone-0035082-g008:**
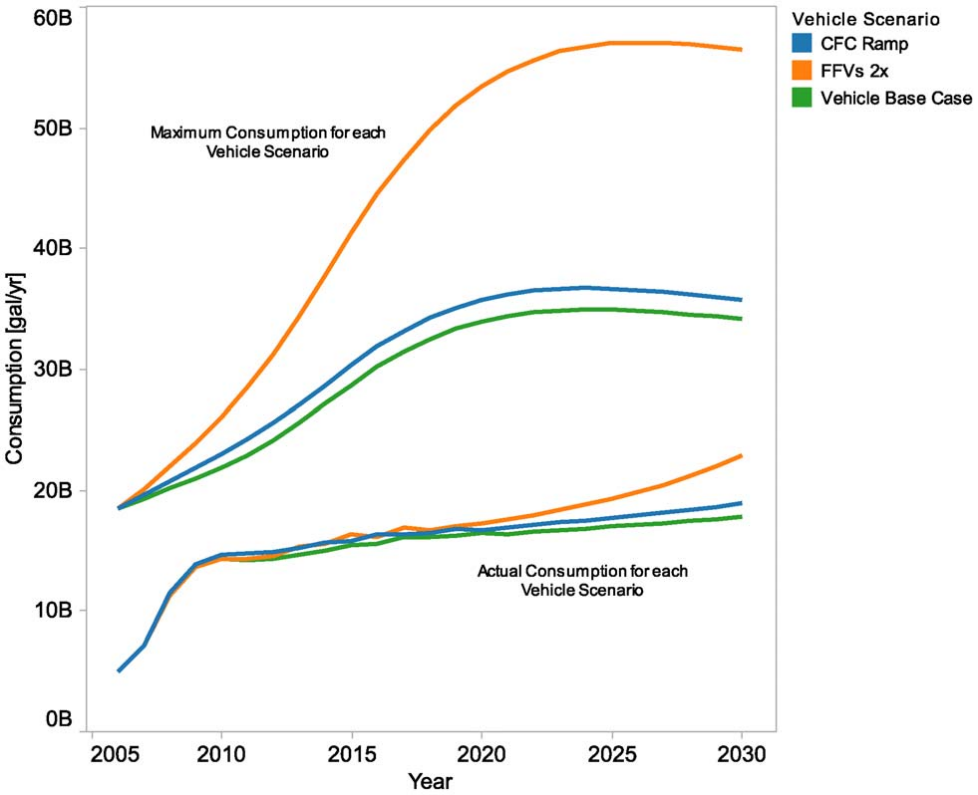
Vehicle scenarios. The figure shows maximum potential ethanol consumption and simulated actual ethanol consumption results for all years under different vehicle scenarios. The Cash for Clunkers ramp scenario (CFC Ramp) increases the rates of retirement of old vehicles and purchase of new vehicles. The flex-fuel vehicles doubling scenario (FFVs 2×) shows ethanol consumption if the share of purchases of FFVs in each time period is assumed to double compared to the base case rate for that period, insofar as possible. FFV penetration alone does not achieve higher ethanol use, but it does lift the maximum potential ethanol consumption and enable higher levels of use when other conditions are favorable.

We explored effects of changes in the base model inputs and assumptions through a series of sensitivity analyses. The sensitivity cases included modifications to assumptions about market actor behaviors, input data about various costs, and vehicle scenario inputs. In Figure 8, we show the effect of different vehicle scenarios on potential and simulated actual ethanol consumption. These vehicle scenarios are not connected to existing or proposed policy, but illustrate the response of the model to hypothetical policy interventions. The gap between potential and simulated actual ethanol consumption reflects that high-blend fuel is not always used by high-blend-capable vehicles, for a variety of reasons.

The Cash for Clunkers ramp scenario (CFC Ramp) illustrates the effect of ramping in a Cash for Clunkers incentive over time to increase the rates of retirement of old vehicles and purchase of new vehicles. Because the percentage of FFVs is assumed to be greater in new vehicles than in the total fleet, an increase in fleet turnover, such as the CFC Ramp scenario, leads to a greater share of FFVs in the total fleet and a modest increase in potential ethanol consumption relative to the Vehicle Base Case. The FFV doubling scenario (FFVs 2×) shows a more aggressive penetration of FFVs and a correspondingly greater increase in potential consumption, where their share of purchases in each time period is assumed to double compared to Vehicle Base Case rates for that period, insofar as possible. While additional FFV penetration alone does not achieve higher ethanol use, it does lift the maximum potential ethanol consumption and enable higher levels of use when other conditions are favorable.

The base model contains a particular set of assumptions about behaviors or circumstances of various market actors with regard to the risk of using biofuels or investing in biofuel technology. Risk aversion is a key issue for new technologies but can be very difficult to estimate. The assumptions used here are based upon the view that people tend to be risk averse in making large investments. The results highlight the importance of these assumptions, but we do not assert that the quantification of these assumptions is exactly correct.

**Figure 9:**
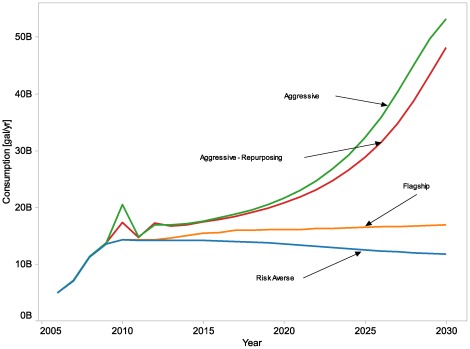
Market assumptions sensitivity. The figure shows ethanol consumption results for all years with differing model assumptions for market actor behavior. The Aggressive cases use two different sets of behavioral assumptions that are more favorable to ethanol consumption, while the Risk Averse case uses a set of behavioral assumptions that is less favorable to ethanol consumption. The Flagship case has the same behavioral assumptions that are used in the rest of the analysis. These sensitivities demonstrate the considerable uncertainty about the behavior of various market players and the substantial effect those behaviors can have on the ethanol market.

In Figure 9, we show market sensitivity cases with base model assumptions adjusted to reflect different levels of risk aversion in market actor behavior. The Flagship case uses the base model assumptions, and the other cases show adjustments to these assumptions as summarized in Table 6. Risk-averse behavior leads to lower ethanol use; aggressive behavior leads to greater ethanol use. These sensitivities represent the considerable uncertainty about the behavior of various market players in response to risk, and the substantial effect those behaviors can have on the ethanol market. If market actors are more risk averse, policy intervention (or favorable market conditions) will result in much less ethanol consumption than if market actors tolerate more risk.

**Table 6:** Summary of Market Assumptions Sensitivity Cases.

Variable or Function Changed →	Occasional UserPrice Response	Regular User Price Response	Station Owner Investment(New Tank)	Station Owner Investment (Repurpose Old Tank)	Conversion Capacity Investment Price Response
**Case Name and Run ↓**					
**Aggressive (7009)**	Base	Base	Aggressive	Base	Aggressive
**Aggressive – Repurposing (7006)**	Base	Base	Base	Aggressive	Base
**Flagship (6901)**	Base	Base	Base	Base	Base
**Risk Averse (6650)**	Risk Averse	Risk Averse	Risk Averse	Risk Averse	Risk Averse

This table shows how the different market actor behaviors were combined into four different cases. Case Name corresponds to the names used in Figure 9, and Run refers to the run number, which may be used to view run results in the supporting information file (Data S1). Occasional User Price Response and Regular User Price Response represent consumer behaviors that address how occasional ethanol users and regular ethanol users responded to ethanol price. The model variable names are “Occasional User Price Attractiveness Weighting” and “Regular User Price Attractiveness Weighting.” Station Owner Investment (New Tank) and Station Owner Investment (Repurpose Old Tank) represent dispensing station owner behaviors that reflect the likelihood that a station owner would invest in a new tank or in repurposing an old tank in order to offer high-blend. In the model, these are two functions called “Investment ‘Hit Rate’” and “Investment ‘Hit Rate’ (RP)” for new and repurposed tankage, respectively. Conversion Capacity Investment Price Response represents conversion capacity investment behavior, measuring the responsiveness of upstream investment to ethanol price. In the model, this is called, “Impact of Price on Cellulosic Growth Rate.”

Five different market actor behaviors were found to have substantial effects. Two of these were consumer behaviors (model variable names are “Occasional User Price Attractiveness Weighting” and “Regular User Price Attractiveness Weighting”) that address how occasional ethanol users and regular ethanol users responded to ethanol price. Two were dispensing station owner behaviors (in the model, these are two functions called “Investment ‘Hit Rate’” and “Investment ‘Hit Rate’ (RP)” for new and repurposed tankage, respectively) that address the likelihood that a station owner would invest in a new tank or in repurposing an old tank in order to offer high-blend. One addresses conversion capacity investment behavior (in the model, this is a function called “Impact of Price on Cellulosic Growth Rate.”), measuring the responsiveness of upstream investment to ethanol price.

**Table 7:** Summary of Insights and Conclusions, along with Mitigating Conditions.

System Property	Key Supporting Factors	Contradicting Conditions
Ongoing favorable market conditions, created bysome combination of high-blend point-of-usesubsidy and incremental gasoline cost, are neededfor sustained use of high levels of ethanol.	Market actors desire continual financial benefitrelative to gasoline to produce, provide, anduse fuel.	If ethanol costs turn out to be lower relative togasoline costs than we assume, or if consumerswere more willing to pay extra for non-gasoline fuelwith less financial benefit, demand wouldbe greater.
Substantial incentives are needed forinfrastructure investment in distribution anddispensing to occur.	Assumed cost of capital and expectedreturn on infrastructure investment limit interestin these investments.	Lower cost of capital and greater expectedreturn on infrastructure investment wouldincrease investment
An incremental gasoline cost can more readilysubstitute for market incentives than forinfrastructure investment incentives. (Incremental gasoline cost might occur without governmentintervention under some market conditions.)	An incremental gasoline cost helps establish a sustained difference between gasoline costand ethanol cost, which is necessary for theethanol market to grow and persist.	An incremental gasoline cost would substitute lesswell for market incentives if it were notpassed up the supply chain to provide market incentives to dispensing stations, distributors, andproducers. It could substitute for infrastructure investment incentives if it affected cost of capital and expected return on investment.
Incentives are most effective when appliedat several points in the supply chain.	Multiple bottlenecks occur across thesupply chain.	If several bottlenecks were resolved through technological or other approaches, fewerincentives would be needed.
Levels of ethanol market targeted in EISAgoals penetration would require tens of billionsof dollars per year in incentives (Figure 5).	Both infrastructure incentives and ongoingmarket incentives are needed to develop andsustain an ethanol market, and theseincentives must be set at certain levels toovercome challenges.	If the challenges to infrastructure and marketgrowth are easier to overcome than estimated,either due to behavior or cost differencesbetween modeling assumptions and reality, cost ofincentives would decline.
The Annual Energy Outlook reference vehiclescenario would not have sufficient FFVs toachieve EISA goals.	If all FFVs in the reference vehicle scenarioalways used high-blend fuel, ethanol usagewould achieve the goal, but 100% useis highly unlikely.	High availability of high-blend fuel and strongmarket incentives (e.g., relatively low price) could increase its usage in FFVs. Alternatively, greaterpenetration of FFVs could helpreach the goal.

The table summarizes insights that can be gained from the analysis, identifies the factors that support the validity of each insight, and conditions that might cause each insight not to hold. Analytic results are best understood as conditional, and this table summarizes the conditions under which insights of the study are likely to be valid.

The four different cases are constructed from combinations of these varying levels of risk aversion in market actor behavior. The “Risk Averse” case is the combination of greater risk aversion, relative to the base model, across all five market behaviors and assumes that access of FFVs to high-blend fuel is worse than in the base model. (In the model, this is a function called “Frac HiBlend Capable with Station Coverage.”) The “Flagship” case represents the same level of risk aversion as found in the base model used in the policy analysis. The “Aggressive-Repurposing” case is an aggressive case for repurposing, in which station owners are more likely to invest in repurposing than in the base model. The “Aggressive” case assumes that station owners are more likely to invest in new tanks and that conversion capacity investors are more responsive to ethanol price than in the base model. These market sensitivities show how significantly risk and risk perception could affect adoption of high-blend fuel.

### Regional Effects

The Biomass Scenario Model divides the United States into 10 regions, according to the agricultural regions used by the U.S. Department of Agriculture. These regions include so-called Appalachian states, Corn Belt states, Delta states, Lake states, Mountain states, Northeast states, Northern Plains states, Pacific states, Southeast states, and Southern Plains states. This division permits the model to be used to analyze whether a regional strategy, which would target particular regions with significantly higher levels of incentive, could achieve higher ethanol market penetration. For this paper we did not analyze such a strategy, nor did we evaluate whether regional strategies might enhance cost effectiveness. Regional issues merit further analysis using the fully integrated model.

### Results Summary and Future Work

Overall, the results of our downstream analysis show that driving ethanol to high levels of market penetration would require substantial subsidies, if model conditions hold. A high penetration of ethanol requires both infrastructure availability and persistently favorable market conditions across the entire supply chain. Favorable market conditions can be achieved either through subsidies or technological improvements that establish relatively low ethanol fuel prices or through incremental gasoline costs, which could represent either a gasoline tax or market conditions that create relatively high gasoline prices without corresponding high ethanol prices (see Table 2 for base model price conditions). Considerable uncertainty about behavioral and market conditions leads to a range of potential ethanol penetration scenarios. Our results suggest that incentives or altered market conditions must be sustained to ensure long-term market stability. These downstream results necessarily neglect the effect of improvements upstream and thus cannot address how robust technology improvement might reduce the need for incentives.

Future work using the full, end-to-end Biomass Scenario Model will expand on this initial analysis of the downstream portion of the supply chain. These future analyses will further explore what policies and combinations of policies appear most effective at establishing an economically sustainable biofuel industry, improving the effectiveness of upstream subsidies in prompting growth throughout the system, minimizing possible unintended consequences, and reducing the overall cost of incentives. Ongoing development of the Biomass Scenario Model also enables analysis of the cost of infrastructure-compatible (“fungible”) fuels–such as biomass-based gasoline, diesel, and aviation fuel–relative to ethanol along with an assessment of the potential competition between multiple biofuels pathways and products.

### Conclusions

Our analysis addressed several major questions (see Introduction). In answer to these questions, we found that an economically sustainable domestic ethanol fuel distribution, dispensing, and end-use system could develop under conditions of strong policy intervention or significant change from current market conditions. Results suggest that, with sufficient policy intervention (or sufficient market change) across the supply chain, the policy goals could be achieved. When assumptions about consumer behavior and system behavior differ, these conclusions generally persist, although substantially different assumptions affect the level of investment in incentives that would be required to develop and sustain an ethanol fuel system.

Our analysis of the downstream portion of the biofuels supply chain shows that, under the assumptions we used, substantial policy intervention or altered market conditions are required to support development of a sustainable ethanol-fuel supply chain in the United States. The conditions in the downstream system that enable the development of a substantial and sustained ethanol market include:

- Competitive pricing of ethanol (or other biofuel) relative to gasoline;
- Sufficient biofuel producer payment;
- Robust investment in infrastructure across the entire supply chain;
- Widespread use of high-blend fuel in FFVs.

The business-as-usual assumptions do not have these conditions, and a robust and sustained ethanol market does not develop. More favorable market conditions or emergence of competitively priced infrastructure-compatible biofuel could reduce or eliminate the policy intervention necessary, but are beyond the scope of this paper.

A combination of policies encouraging infrastructure investment and policies supporting favorable market conditions appears to be most effective in establishing an economically sustainable, domestic ethanol fuel supply chain. An incremental gasoline cost might occur without government intervention under certain market conditions, or would generate revenue if implemented as a gasoline tax and could partially substitute for other market incentives. Favorable assumptions about the behavior of market actors lead to a lesser need–but do not eliminate the need–for policy intervention or market changes. The Vehicle Base Case scenario is unlikely to achieve the EISA goal using ethanol in light-duty vehicles alone, unless an improbably large share of FFV owners were able to utilize high-blend fuel, but alternative vehicle scenarios with greater FFV penetration might do so. Results of our downstream analysis identify potential issues in achieving existing goals and can inform selection of other goals, considering the system conditions necessary for their achievement and the estimated cost of supporting policies. Key insights and conclusions are shown in Table 7, along with conditions that would support or alter these findings. The modeling work shows (given assumptions):

- Levels and duration of favorable market conditions required for ethanol to penetrate the market;
- Levels of incentives needed to prompt significant infrastructure investment;
- Infrastructure investment is not readily encouraged through point-of-use price differential alone;
- Improved efficacy of coordinated incentives compared to interventions at a single point of the supply chain;
- FFV availability in the AEO vehicle scenario is likely insufficient for ethanol markets to reach EISA goals.

Our analysis of the downstream ethanol supply chain using the Biomass Scenario Model provides preliminary insights regarding both key elements of the downstream vehicle system and how policy incentives could shape the development of sustained and significant ethanol fuel use in the United States to meet policy goals. These insights may be useful in shaping future goals and the policies to achieve them. We are pursuing additional analysis on other parts of the biofuels supply chain and are developing an analysis based on the full end-to-end Biomass Scenario Model.

## Supporting Information

Data S1 **Supporting information is provided in a Microsoft Access Database (2003).** This database contains seven tables. The “Additional_Runs” table provides results for incremental gasoline costs in addition to those used in the figures. The following four tables (Data_Figure_4, Data_Firgure_5&6, Data_Figure_7&8, Data_Figure_9) contain model run results that were used in figures 4 through [Fig pone-0035082-g005] [Fig pone-0035082-g006] [Fig pone-0035082-g007] [Fig pone-0035082-g008] 9. The “EISA” table contains Total Renewable Fuel Requirement data from the regulation implementing EISA (Federal Register Vol. 75, No. 58, Friday, March 26, 2010), and does not represent model results. The “Key_to_Runs_Figures” summarizes which runs were used in which figure. The result provided is national consumption, either actual or maximum, of ethanol or gasoline in flex-fuel or conventional vehicles over 25 years (2006–2030), resulting in 100 records per run. (Actual and maximum gasoline consumption are the same and actual ethanol consumption is not divided by vehicle type.) The supplemental data is an aggregation and summary of a much larger database that retains additional metrics besides national consumption from each run, as well as data on runs not included.
(ZIP)
